# Organosilane and Polyethylene Glycol Functionalized Magnetic Mesoporous Silica Nanoparticles as Carriers for CpG Immunotherapy *In Vitro* and *In Vivo*

**DOI:** 10.1371/journal.pone.0140265

**Published:** 2015-10-09

**Authors:** Hengrui Zheng, Songsong Wen, Yang Zhang, Zhenliang Sun

**Affiliations:** 1 Center for Medical Research, the Affiliated Hospital of Qingdao University, Qingdao, 266003, China; 2 Fengxian Hospital affiliated to Southern Medical University, 6600 NanFeng Road, Shanghai, 201499, China; 3 Qilu Pharmaceutical Co. Ltd, Jinan, 250101, China; 4 Tong Ren Hospital Shanghai Jiao Tong University School of Medicine, 1111 XianXia Road, Shanghai, 200336, China; Tufts University, UNITED STATES

## Abstract

Cytosine–guanine (CpG) containing oligodeoxynucleotides (ODN) have significant clinical potential as immunotherapeutics. However, limitations exist due to their transient biological stability *in vivo*, lack of specificity for target cells, and poor cellular uptake. To address these issues, we prepared amine magnetic mesoporous silica nanoparticles (M-MSN-A) then further modified with polyethylene glycol (PEG) for use as CpG delivery vectors. The PEG modified M-MSN-A (M-MSN-P) had notable CpG ODN loading capacity, negligible cytotoxicity, and were easily internalized into cells where they released the loaded CpG into the cytoplasm. As a result, such complexes were effective in activating macrophages and inhibiting tumor cells when combined with chemotherapeutics *in vitro*. Furthermore, these complexes had excellent immuno-stimulating activity *in vivo*, compared to the free CpG therapeutics. We report here a highly effective MSNs-based delivery system with great potential as a therapeutic CpG formulation in cancer immunotherapy.

## Introduction

Cytosine–guanine (CpG) containing oligodeoxynucleotides (ODN) are attractive as potentially effective immunotherapeutic agents. In human and animals, CpG ODN sequences can be recognized as ‘danger’ signals by the immune system, resulting in stimulation of both the innate and adaptive immune responses [1–3]. The bioactivity of the CpG ODN sequences has been attributed to unmethylated CpG nucleotides flanked by specific bases [4, 5]. Currently, clinical trials are underway to evaluate CPG ODN sequences as therapeutic agents and vaccine adjuvants; their efficacy in the treatment of malignant cancer as well as infectious and allergic diseases is also being tested [6–8].

Despite these clinical trials, the use of free CpG ODN still presents several difficulties due to limitations in biological stability *in vivo*, unfavorable biodistribution characteristics, lack of specificity for target cells and poor cellular uptake [9, 10]. To overcome these issues, researchers are developing new formulations and delivery systems, including lipid-based delivery systems that represent one of the most advanced drug delivery technologies [11, 12]. However, the *in vivo* use of lipid-based delivery systems is limited, in part, due to a large, heterogeneous size distribution. Additionally, lipid-based delivery system are characterized by non-specific interactions with cells, proteins and other macromolecules in the circulation [13, 14], and a range of adverse side effects all of which can result in lethality [15, 16].

Mesoporous silica nanoparticles (MSNs) have emerged as one of the most appealing candidates for delivering a variety of drugs such as proteins, anti-cancer chemicals and plasmid DNA vectors [17–19]. These silica-based nanoparticles exhibit several characteristics that could be beneficial for the delivery of therapeutic agents, including a large surface area, an ordered pore structure and a modifiable surface [19–21]. Previous studies have also demonstrated that MSNs possess excellent biocompatibility and can be degraded and metabolized in a relatively short period of time *in vivo* [22, 23]. To the best of our knowledge, there are few data regarding the delivery of CpG ODN with MSNs or their exploitation as immunotherapeutic agents, particularly *in vivo*. Recently, Zhu and his co-workers have tried to deliver CpG drugs by using some mesoporous silica large particles (with the diameter of around 500 nm), such as SBA-15, hollow silica particles and obtained some achievement [24, 25]. These investigations thus provide us with clues to engineer effective MSNs-based CpG delivery system for cancer immunotherapy.

In this study, we designed a drug delivery system based on amine magnetic mesoporous silica nanoparticles (M-MSNs) that were further modified with polyethylene glycol (PEG). This kind of nanopartilces not only possesses sub-100 nm sizes which meet the requirements of *in vivo* applications, but also consists of the magnetic nanoparticle core. In this sense, the magnetic core provides magnetically targeted guidance by applying an external magnetic field. Target delivery allows drugs to be locally directed and concentrated near the action sites under magnetic guidance, and the particles can be removed when the therapy is completed [26, 27]. Herein, this magnetic CpG system may be a more promising candidate for facilitating drug cellular uptake *in vitro* and target delivery *in vivo*. Here, we emphatically discuss its CpG adsorption/desorption behaviors, transfection mechanism as well as the immunostimulatory activities.

## Materials and Methods

### Materials

All reagent-grade chemicals were used as received. Millipore water (18.2 MΩ·cm) was used in the preparation of all aqueous solutions. Tetraethyl orthosilicate (TEOS, AR) and ammonium nitrate (NH_4_NO_3_, AR) were purchased from Aladdin. 1, 3, 5-trimethyl-benzene (TMB, 99%) was purchased from ACROS. Ethylene glycol (EG, used as dispersing agent), methoxy poly (ethylene glycol) succinimidyl glutarate (mPEG-SG, MW 2000, 99%, used as PEG modification agent) was purchased from Biomatrik Inc (Jiaxing, China). Fluorescein isothiocyanate isomer I (FITC, 95%) was obtained from Sangon Biotech (Shanghai) Co., Ltd. Cetyltrimethylammonium bromide (CTAB, AR), 3-aminopropyltriethoxysilane (APTES, AR), NH_4_OH (25 wt%, AR), ethylene glycol (AR) and absolute ethanol (AR) were purchased from Sinopharm Chemical Reagent Co., Ltd., China. RPMI 1640 medium, phosphate buffered saline (PBS), and fetal bovine serum (FBS) was obtained from Gibco (USA). Oligodeoxynucleotides containing the unmethylated bacterial CpG motif with a phosphorothioate backbone (ODN 1826), ODN 1826 conjugated with cyanine dye (Cy3) and control oligodeoxynucleotides with an inverted CpG motif (ODN 1720) were purchased from Sangon (Shanghai, China). The sequences were as follows: ODN 1826, 5’-TCC ATG ACG TTC CTG ACG TT-3’; ODN 1720, 5’ -TCC ATG AGC TTC CTGATG CT-3’; ODN 1826 (denoted as CpG), ODN 1720 (denoted as Non-CpG). The oligodeoxynucleotides were dissolved in endotoxin-free sterile distilled deionized H_2_O (ddH_2_O) according to the manufacturer’s recommendations and were used at the indicated concentrations. Dimethyl sulfoxide (DMSO), 3-(4, 5-dimethylthiahiazol-2-y1)-2, 5-di-phenyltetrazolium bromide (MTT) were obtained from Sigma-Aldrich (Germany). The gene transfection kit (Lipofectamine-2000, used as positive control) was purchased from Invitrogen (USA); The 4’-6-diamidino-2-phenylin- dole (DAPI) and LysoTracker Red were purchased from Beyotime Institute of Biotechnology. Doxorubicin hydrochloride (denoted as DOX) was obtained from Sigma (USA). Transwell plates with a pore size of 0.4 μm and 12 mm in diameter were purchased from Corning (USA). A TNF-α ELISA kit (MTA00B) and Mouse IL-12p70 ELISA kit were purchased from Shanghai ExCell Biology, Inc. 29G-needle syringes was obtained from BD (USA).

### Ethics statement

All the procedures were in strict accordance with the PR China legislation on the use and care of laboratory animals and with the guidelines established by the Institute for Experimental Animals of Shanghai Jiao Tong University, and were approved by the research ethics committee of Shanghai Jiao Tong University for animal experiments.

### Cell cultures and animals

Hela cells and the RAW264.7 murine macrophage-like cell line were grown in RPMI 1640 medium supplemented with 10% heat-inactivated FBS, 100 units/ml penicillin, 1000 μg/ml streptomycin. Five-week-old male BALB/c mice (20±2 g) were purchased from B&K Universal Group Limited, Shanghai, China. Animals were maintained under conventional housing conditions in isolated cages with a 12 h light/dark cycle at constant temperatures (24–26°C) and free access to food and water.

### Preparation and characterization of amine and further PEG functionalized M-MSNs

Amine functionalized magnetic mesoporous silica manoparticles (M-MSNs) were synthesized as previously reported, with some modifications [28]. Briefly, oleic acid stabilized magnetic Fe_3_O_4_ nanoparticles (MNPs) were prepared via a coprecipitation method and then dispersed in chloroform at a concentration of 6.0 mg Fe/ml [29]. Then, 0.74 ml of the suspension was added to 5 ml of an aqueous solution containing 0.15 g CTAB with continuous ultrasonication at 50°C for 30 min. Further, the mixture was heated to 70°C and aged for 15 min under stirring to evaporate the residual chloroform. After evaporation of the chloroform, a transparent black dispersion was obtained and added into a solution composed of 35 ml water, 10 ml EG, and 0.7 ml NH_4_OH (25 wt%). Afterward, 0.44 ml TMB as a pore-swelling agent was added to the dispersion at 70°C and homogenized by continuous stirring for 2 h. The sequential addition of 0.45 ml TEOS and 0.05 ml APTES in a drop wise manner completed the reaction solution. All the reagents were then stirred at 70°C with refluxing for 3 h. The resulting products were collected by centrifugation (1000 rpm, 30 min) and washed with ethanol and water three times. Finally, the templates were removed by a highly efficient ion-exchange method. In order to remove the template completely, the nanoparticles were dispersed in a 60 ml ethanol solution containing 60 mg NH_4_NO_3_ and ultrasonicated in a water bath for 2 h. The procedure was repeated three times. Based on the results of previous literatures [30, 31], these operations can ensure the complete removal of the template. The resulting amine functionalized nanoparticles are denoted as M-MSN-A.

Modification of the M-MSN-A was achieved by mixing it with mPEG-SG and ethanol in a ratio of 1 mg M-MSN-A: 1 mg mPEG-SG: 1 ml ethanol. The mixture was shaken for 3 h at room temperature to form covalent bonds between the amine groups on the outer surface of M-MSN-A and the succinimidyl groups of mPEG-SG [32]. The products were collected by centrifugation and then washed with ethanol three times to remove any unreacted mPEG-SG, The obtained PEG modified nanoparticles are denoted as M-MSN-P.

To generate FITC-labeled samples, 30 mg nanoparticles (M-MSN-P) were reacted with 0.5 mg FITC in a 5 ml ethanol solution under dark conditions overnight. Samples were then centrifuged and washed with ethanol three times to remove any unreacted FITC. The FITC-labeled nanoparticles are denoted as F-M-MSN-P.

Transmission electron microscopy (TEM) images were captured on a JEM 2010 (JEOL, Japan) instrument with 200 kV accelerated voltage. Nitrogen sorption isotherm was measured with a Micromeritics ASAP2010 analyzer (USA) at 77 K. Before measurements, the sample was dried in a vacuum oven at 373 K for 6 h, and outgassed in the instrument at 373 K to a residual pressure below 6.65×10^−6^ bar. The pore size distribution was derived from the desorption branch of the isotherm using the NLDFT method and the Quantachrome Autosorb I software (Quantachrome Instruments, USA) [33]. The thermal behavior was characterized using a thermogravimetric analyzer (DTG-60/60H, Shimadzu Ltd., Japan) with a heating rate of 10°C/min under air atmosphere. The hydrodynamic diameter distribution and the Zeta potential of different samples were performed using the Dynamic Lighter Scattering (DLS) method on a Zetasizer Nano instrument (Malvern, UK) at 298 K.

### CpG adsorption

A series of CpG solutions with concentrations ranging from 62.5 to 650 μg/ml was prepared by dissolving different amounts of CpG in distilled water. Then 0.2 mg of M-MSNs or M-MSN-P was mixed with 200 μl of each CpG ODN solution in 2 ml centrifuge tubes. The resulting mixtures were shaken continuously in a shaking bath with a speed of 200 shakes min^-1^ at 25°C for 3 h. The amount of CpG in the supernatant was measured by using a NANODROP 1000 spectrophotometer (Thermo Scientific, USA). The difference in the CpG ODN amount in solution before and after adsorption was determined as the amount of CpG adsorbed on the particles. For the release behaviors, the saturated M-MSN-A/P (0.2 mg) loading CpG were dispersed in 400 μl ddH_2_O or PBS in 2 ml centrifuge tube. Then, the tube was placed in an air shaker bath at 100 rpm/min (at 37°C) for *in vitro* release. At scheduled time, samples were centrifugally separated for 1 min and 50 μl of the supernatant was replaced with the same volume of fresh ddH_2_O or PBS (pre-warmed to 37°C). The amount of CpG presented in the supernatant was determined by using NANODROP 1000 spectrophotometer as mention above.

### Cell uptake assay

CpG uptake was carried out in 24-well cell culture plates. RAW264.7 cells were seeded in plates at 5×10^4^ cells/well and allowed to attach overnight. The M-MSN-P particles loading CpG (conjugated with Cy-3) were diluted in distilled deionized H_2_O (ddH_2_O) and dispersed in RPMI 1640 medium supplemented with 10% FBS. Subsequently, 200 μl of the resulting mixture was added to each well. The CpG dosage of all the experimental groups including the group of bare CpG molecules (denoted as free CpG group), CpG loaded in Lipofectamine agents (denoted as Lipofectamine group), CpG loaded in M-MSN-P (denoted as M-MSN-P group), was fixed to 15 μg/ml. While, unmodified M-MSNs can’t be able to absorb the CpG, so M-MSNs group is conducted by the same amount of M-MSNs particles as M-MSN-P (e.g. 0.17 mg particles per ml culture medium). M-MSNs particles also experienced the same adsorption experiments (actually, there is no CpG loaded). When particles/cells incubated at 37°C, a neodymium-iron-boron (ND-Fe-B) permanent magnet was placed under the plates for 1 h to promote the cellular uptake of the delivery vehicles (The picture of specific magnet for 24-well cell culture plate is shown in Figure A in S1 Text). Then the magnet was removed and the particles/cells were incubated for another 2 h. Cells were then washed 2 times with PBS and fixed with 4% paraformaldehyde in PBS at 25°C for 30 min followed by a third washing with PBS prior to draining the liquid. The cells were viewed under a Nikon TE2000-U inverse fluorescent microscope after staining their nuclei with 200 μl of a DAPI solution.

Confocal laser scanning microscopy (CLSM, Leica SP5 II, Germany) was used to assess the intracellular trafficking of the loaded nanoparticles. RAW264.7 cells were plated into glass-bottom dishes at a density of 3×10^4^ cells per dish. After incubation at 37°C for 24 h, the growth medium was replaced with fresh medium containing F-M-MSN-P vehicle. After 3 h of the same magnetic/non-magnetic incubation, cells were treated with LysoTracker Red for 30 min and DAPI for 5 min and then captured by CLSM.

### Cytotoxic and TNF-α secretion assays using RAW264.7 cells

The MTT assay was employed to determine the cytotoxicity of functionalized M-MSNs in the macrophage cell line. RAW264.7 cells (200 μl) were seeded in 24 well plates at 2 × 10^5^ cells/ml in RPMI 1640 medium. After 24 h incubation, the medium was abandoned and the cells were washed with 0.2 ml PBS for three times, CpG loaded M-MSNs or M-MSN-P in fresh medium were added to the cells and keep on incubating for 8 h. The supernatant was collected the measuring the TNF-α secretion (using a TNF-α ELISA kit, MTA00B) and then replaced with 200 μl 0.5 mg/ml MTT in RPMI 1640 medium without FBS. After 4 h of incubation at 37°C, the medium was discarded and the precipitation in the cells was dissolved by DMSO (150 μl/well) then, the dissolvable solution was shaken for 10 min. Plates were ultimately read on a microplate reader at 570 nm. The data reported were the mean of three examinations [34].

### Anti-proliferative activity of CpG loaded particles on tumor cells co-cultured with RAW264.7 cells

Transwell plates were utilized to determine the anti-tumor effect of CpG. In brief, RAW264.7 (4×10^4^ cells/well) and Hela cells (4×10^3^ cells/well) were placed in the upper and lower chambers, respectively. Free CpG and CpG loaded particles were added to the upper side at a final concentration of 10 μg CpG/ml. DOX was also added to the upper side at a final concentration of 1 μg DOX/ml for the CpG+DOX treatment groups. After 48 h incubation at 37°C, the proliferative capacity of Hela cells in the lower chambers was measured using the MTT assay as described above.

### IL-12 production in mice

Free CpG or CpG loaded M-MSN-P suspended in a 0.9% NaCl aqueous solution (25 and 50 μg/mouse for two groups, respectively) was injected into mice through the tail vein using a 29G-needle syringe. Six hours later, the mice were anesthetized and their blood was collected from the vena cava. Then the serum was obtained after centrifugation at 3000 g for 20 min at 4°C. The concentration of IL-2 In the resulting serum was measured using a Mouse IL-12p70 ELISA kit (Shanghai ExCell Biology, Inc).

### Statistical analysis

Each group of raw data was analyzed statistically using one way analysis of variance (ANOVA) by virtue of GraphPad Instat software (version 3.0, GraphPad Software, Inc., USA). Subsequently, statistical differences in each group were tested using student *t*-test (with *P* < 0.05 considered as statistically significant).

## Results and Discussions

### Synthesis of modified M-MSNs

**Fig 1:**
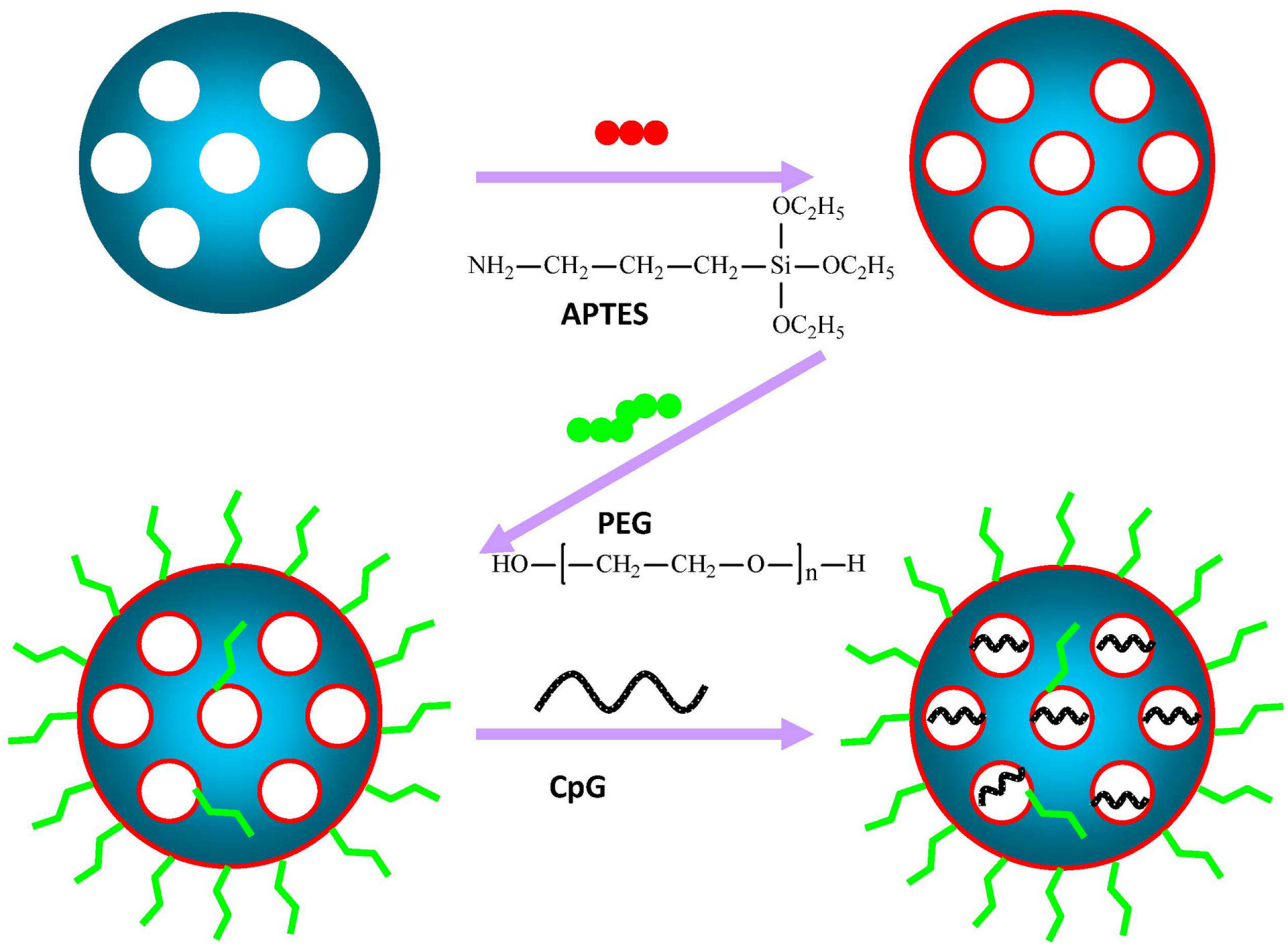
Schematic illustration of the steps for the amino-modification (M-MSN-A) and PEG (MW 2000) grafting (M-MSN-P) as well as CpG loading into the particles.

**Fig 2:**
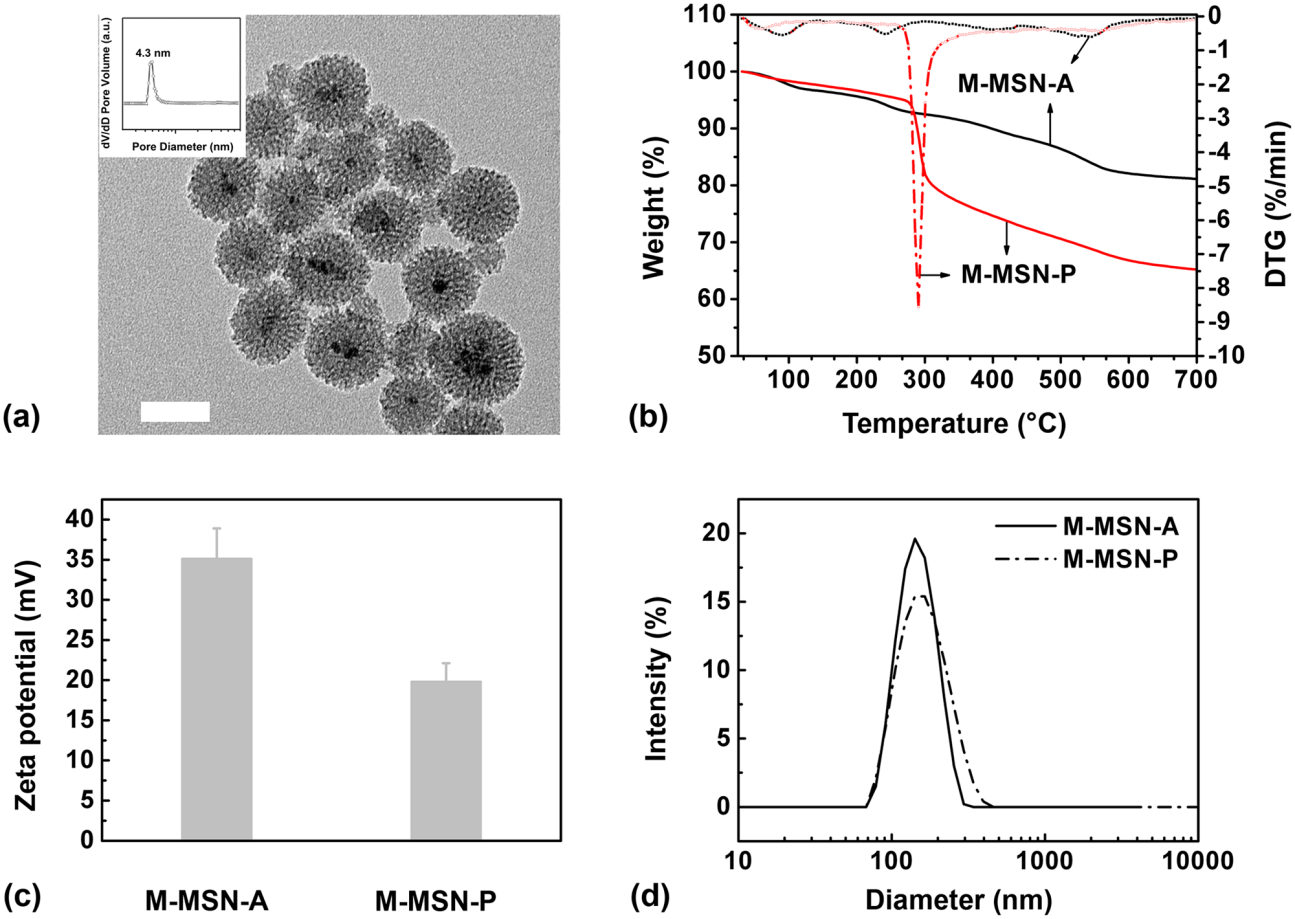
Amine functionalized nanoparticles are denoted as M-MSN-A. Following further PEGylation, they are denoted as M-MSN-P. (a) TEM micrograph for M-MSN-A. The bar represents 50 nm. The inset shows the pore size distribution plot for M-MSN-A. (b) TG and DTG curves, (c) Zeta potential and (d) particle size distributions of M-MSN-A and M-MSN-P.

Surface modification of mesoporous silica is an essential requirement for transferring DNA/RNA. We synthesized M-MSNs with positive charges on the silica surface, which in turn permitted electrostatic interactions with negatively charged CpG ODN by using APTES (the resulting nanoparticles are denoted as M-MSN-A). To deal with the complex *in vivo* environments and reduce unspecific interactions with proteins, M-MSN-A were further PEGylated (M-MSN-P) [35]. The procedure employed for the functionalization and loading of CpG is illustrated in Fig 1. The characterizations of modified M-MSNs are displayed in Fig 2.

A representative TEM image demonstrates a typical pore size distribution of the M-MSN-A, having a uniform and discrete spherical shape with a mean diameter of 50±15 nm, as shown in Fig 2(a). Cylindrical mesopores (~4.3 nm in diameter) were observed in a radical arrangement within the shell layer. Most nanoparticles contained a single magnetic Fe_3_O_4_ nanoparticle (MNP, ca. 12 nm in size) core in the center of the mesoporous silica shell with an average thickness of 20 nm. The M-MSN-P exhibited similar morphologies to the M-MSN-A (data not shown). The TG curve in Fig 2(b) indicates a PEG grafting amount of 17.5%. PEGylation led to a lower Zeta potential (~20 mV) compared with that of M-MSN-A (~35 mV) as shown in Fig 2c (measured in 20 mM phosphate buffer, pH 7.0, the underlying data are shown in Table A in S1 Text). PEGylation resulted in a slightly larger particle size compared with the M-MSN-A as determined by DLS and shown in Fig 2(d) (Table B in S1 Text).

### CpG adsorption

**Fig 3:**
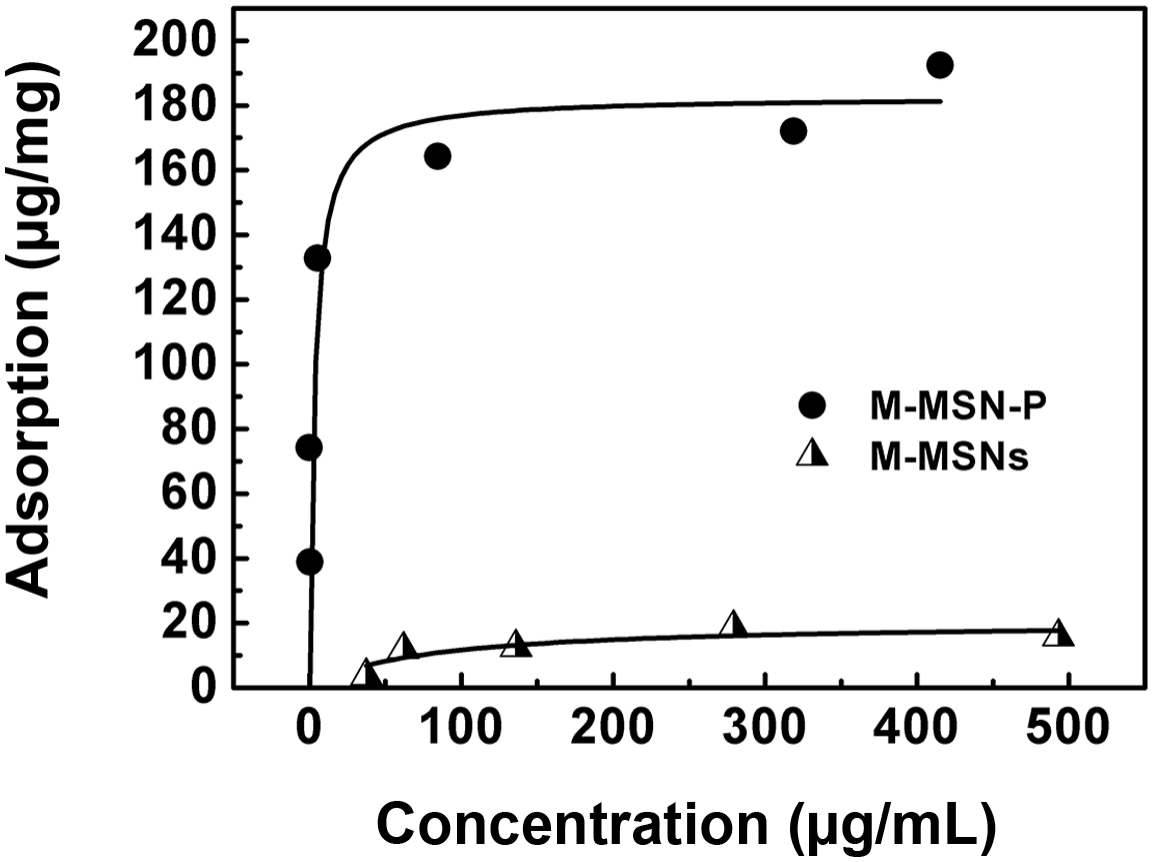
CpG adsorption against M-MSN-P and M-MSNs.

After the functionalized M-MSNs were obtained, their affinity to CpG ODN was measured. The M-MSNs (without any modification) were used as a control group. As shown in Fig 3, the saturated adsorption capacity of M-MSN-P is 180 μg/mg particles (please see Table C in S1 Text for underlying data); this value is almost 7 times higher than the functionalized MSNs with large mesopores (13~24 nm) reported previously by Qiao and co-workers [36]. Likely, the size matching between the pore size of M-MSN-P (~4 nm) and CpG (~2 nm) contributed to this significant difference [37–39]. Further, since the interaction of CpG with functionalized silica particles is electrostatic, the surface area of the particles may be another important factor to influence the loading capacity.

### Cytotoxicity study

**Fig 4:**
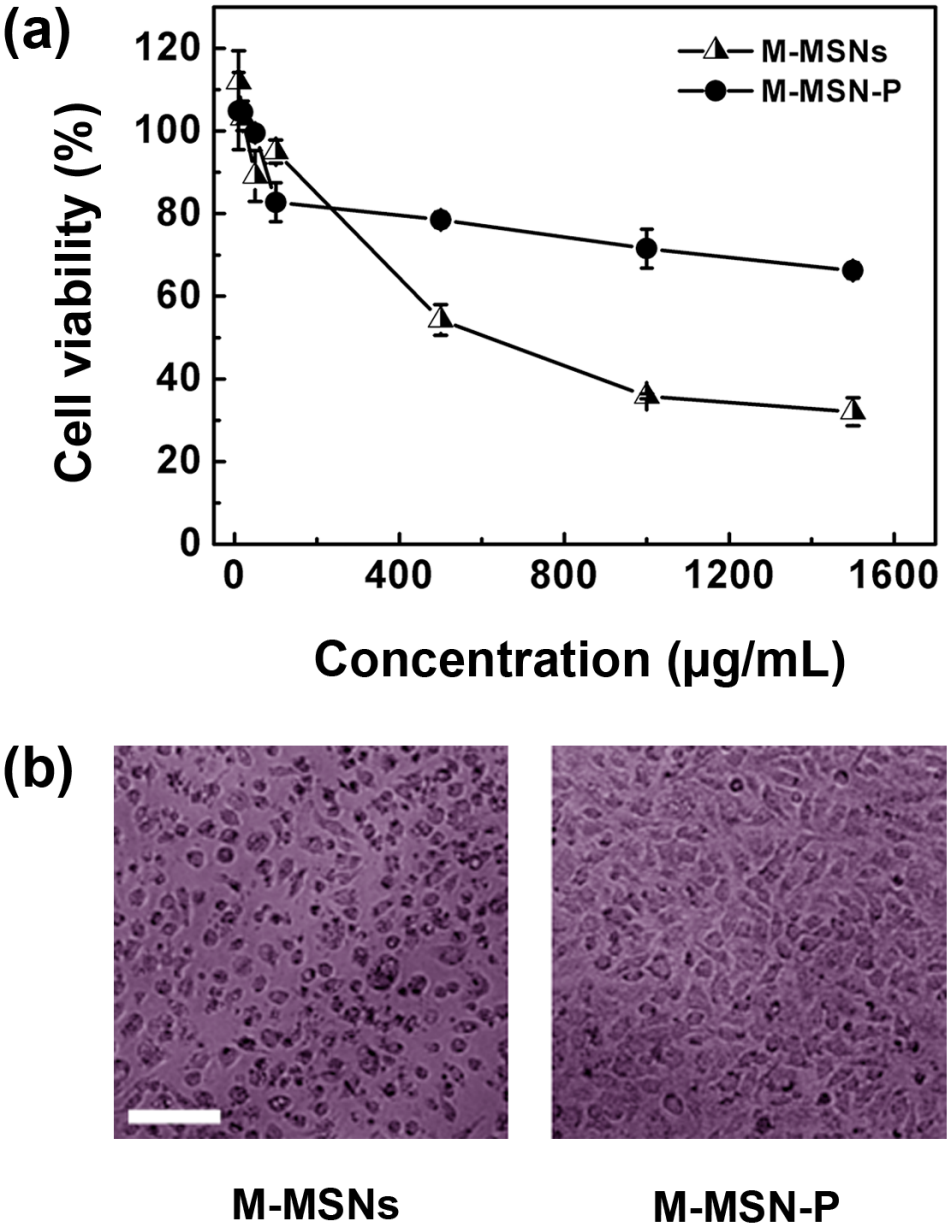
Cytotoxicity of M-MSNs and its functionalized counterpart. (a) The curves show the MTT cell viability results (reported as a % of the medium-treated (control) cells) after 8 h treatment with different particles doses. (b) Cell images: cytotoxicity comparison between the two particles at a concentration of 500 μg/ml. Bar = 100 μm.

Outstanding biocompatibility properties are an important characteristic for the development of ideal carriers for CpG immunotherapeutics. Therefore, we evaluated the cytotoxicity of the M-MSNs and its functionalized forms as shown in Fig 4 (Table D in S1 Text). Overall toxicity increased with increasing concentrations of nanoparticles (Fig 4(a)), the phenomenon was confirmed in cell images (Fig 4(b)). Specifically, at concentrations greater than 500 μg/ml, all nanoparticles were toxic to some extent; however, at concentrations less than 100 μg/ml, the samples displayed low cytotoxicity. In addition, the M-MSN-P is less toxic than M-MSNs, an effect that may be due to the PEG steric hindrance weakening cell/particle interactions [40].

### Cellular uptake

**Fig 5:**
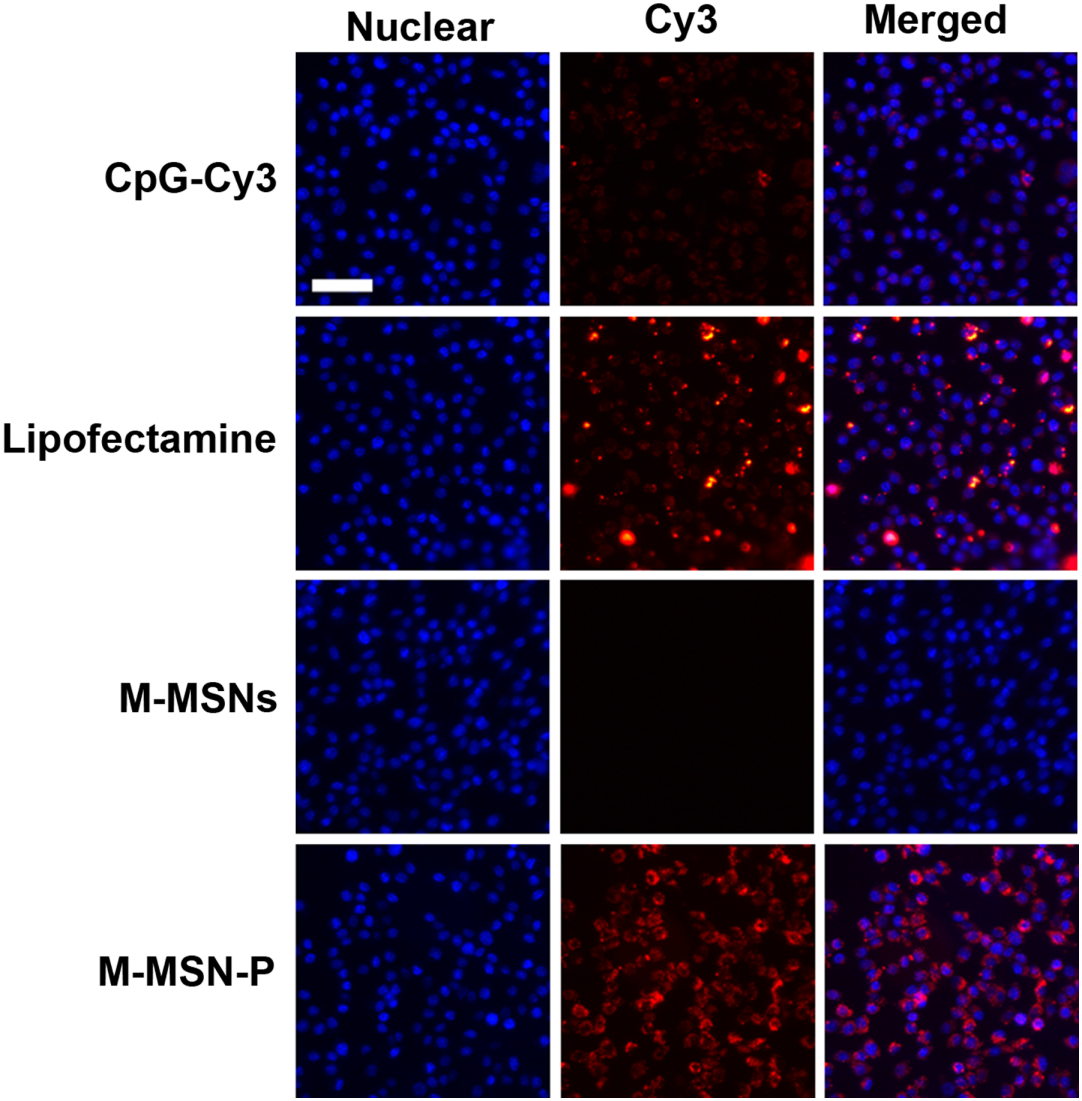
Cell uptake of CpG loaded in M-MSN-P. The cell uptake efficiency was examined by labeling the CpG with Cy3. The free oligo CpG-Cy3 and Lipofectamine-CpG-Cy3 were utilized as controls. M-MSNs are unable to carry CpG-Cy3 thus no Cy3 signal is observed. M-MSN-P with CpG-Cy3 shows a positive signal. Bar = 50 μm. The doses of free CpG or CpG in loaded carriers were equivalent to 15μg/ml.

RAW264.7 macrophage cells were used to study the internalization capacity of CpG loaded in functionalized M-MSNs as illustrated in Fig 5. To adequately follow the internalization process, CpG was labeled with cyanine dye (Cy3). There was no observed Cy3 signal from native M-MSNs and only a weak signal from the free CpG group. Cells treated with Lipofectamine/CpG complexes demonstrated a more profound Cy3 signal as well as the formation of large aggregates when compared to the free CpG group. This is in agreement with the previous reports on lipid-based delivery systems, as mentioned above [13, 14]. In contrast, the modified M-MSNs (M-MSN-P)/CpG demonstrated the highest intensity of Cy3 signaling that was distributed equally throughout the cells compared to both the free CpG and Lipofectamine/CpG complex groups. Further to confirm the above results, we supplemented another experiment displayed in S1 Text. Flow cytometry analysis (BD FACSAria II, US) in Figure B in S1 Text revealed that M-MSN-P loading CpG resulted in a clear shift in the MFI (mean fluorescent intensity), compared with M-MSNs and free CpG (without any vectors) groups. These results provided the envidence that M-MSN-P performed better than the unmodified particles (M-MSNs) groups and free CpG groups.

### Mechanism of CpG transfection

**Fig 6:**
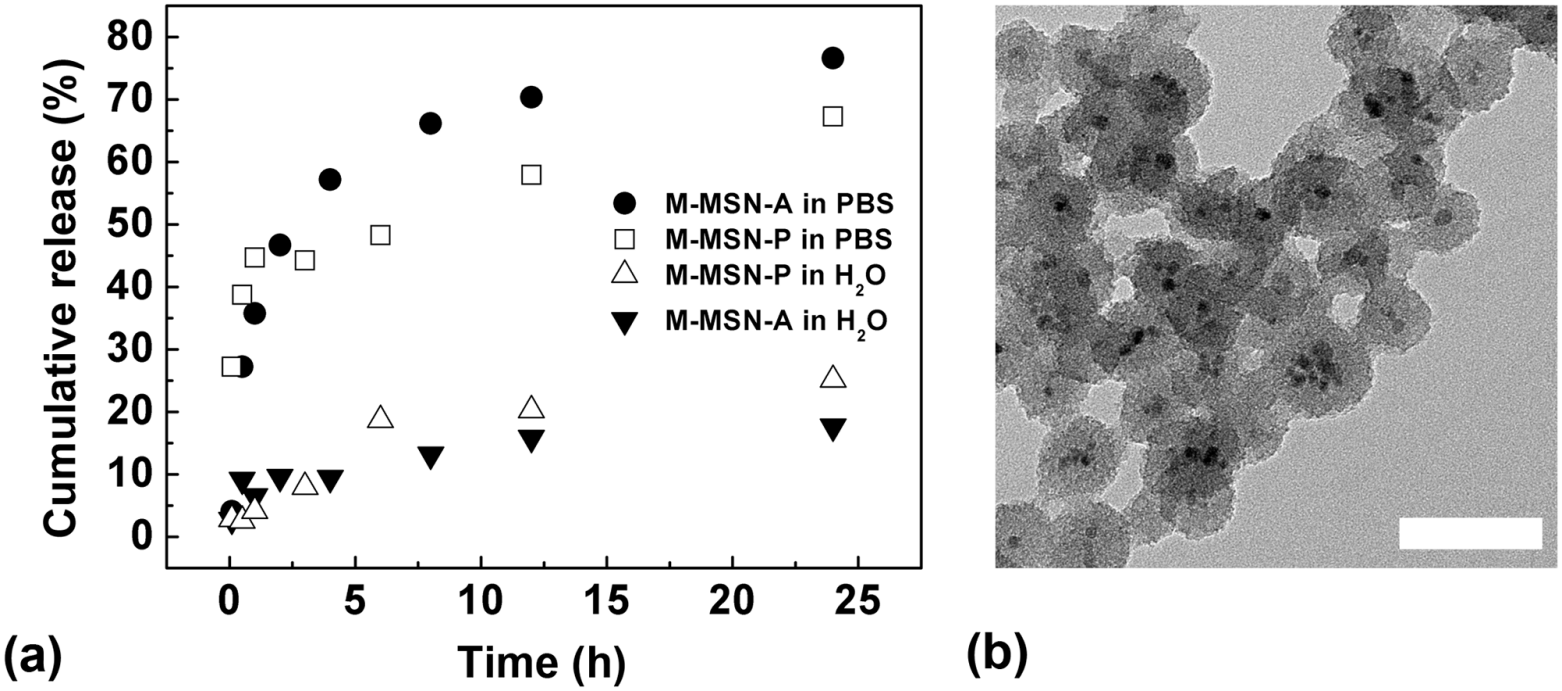
(a) Profiles of the cumulative release of CpG from M-MSN-A and M-MSN-P. (b) TEM micrographs of M-MSN-P-CpG after 8 h immersion in PBS, bar = 100 nm.

**Fig 7:**
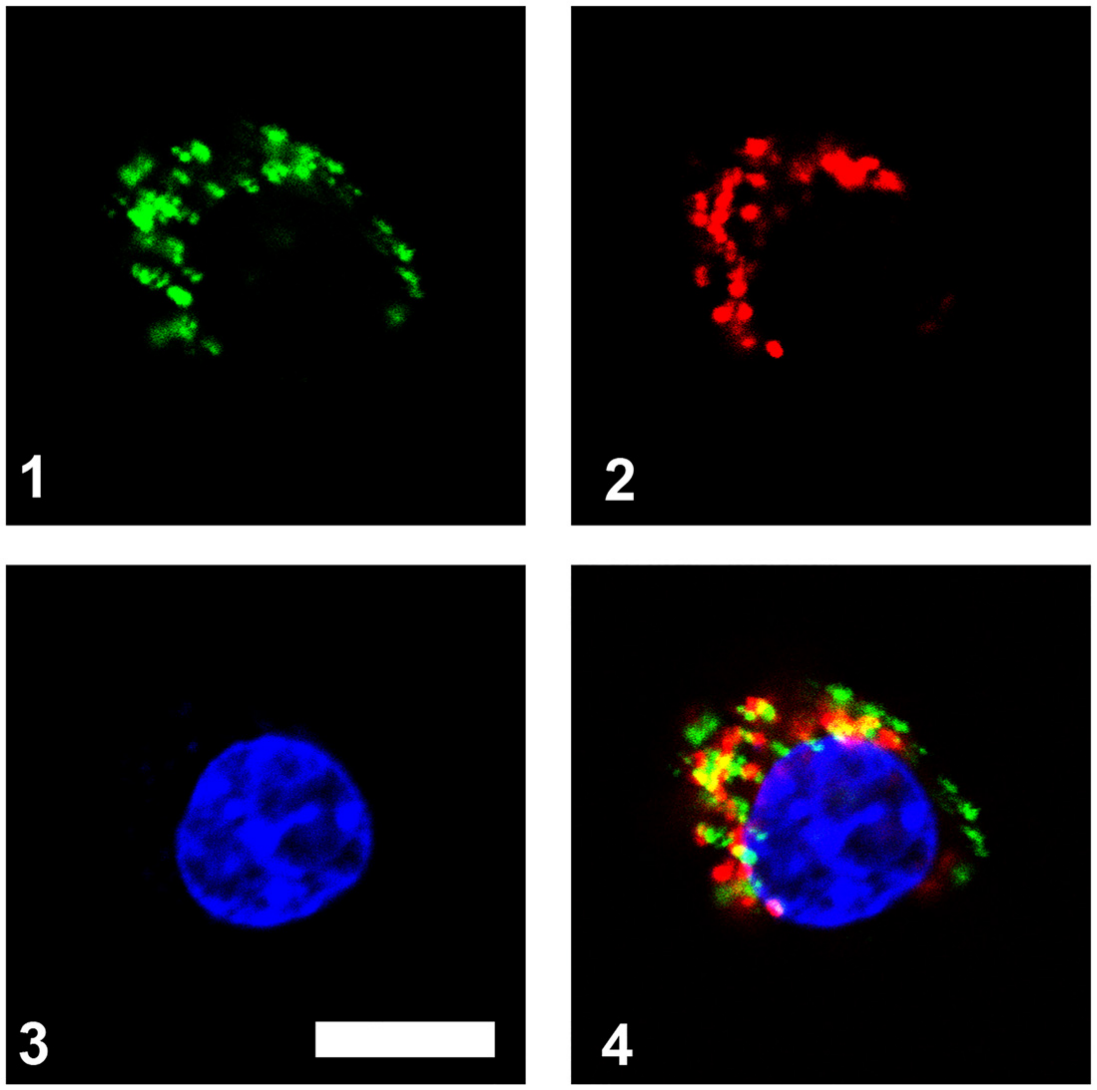
Confocal laser scanning microscopic images of RAW264.7 cells incubated with M-MSN-P-CpG nanoparticles for 3 h. (1) The particle is labeled with fluorescein isothiocyanate (FITC, emission of green fluorescence), (2) the endolysosomes in cells are stained with LysoTrackerRed (emission of red fluorescence), (3) the nuclei are stained with 4,6-diamino-2-phenyl indole (DAPI, emission of blue fluorescence), bar = 10 ìm, (4) the overlapped image of 1–3.

RAW264.7 macrophage cells possess an inherently active phagocytic capcity; therefore, the free CpG group still presented a weak signal (Fig 5). The intracellular uptake of CpG loaded in M-MSN-P is likely due to two factors: 1) CpG extracellular release from vectors in culture medium, and 2) CpG intracellular release from the vectors within cells. In order to verify this inference, we investigated the extracellular release (Fig 6) and intracellular endocytosis behaviors (Fig 7) of M-MSN-P.

As shown in Fig 6(a), both of the complexes release CpG to some extent within the PBS buffer environment and water (Table E in S1 Text). Recent reports suggest that CpG release from functionalized M-MSNs is due to degradation of the nanoparticles’s silica shell [41]. To confirm this, we determined the morphology of the samples released in PBS buffer and water using TEM. Fig 6(b) indicates that the silica shell of M-MSN-P underwent dramatic degradation in PBS buffer within 8 h (for more information, please see the TEM micrographes of M-MSN-P after 1, 8 and 24 h immersion in PBS, as shown in Figure C in S1 Text). M-MSN-A displayed similar degradation morphology after 8 h incubation in PBS. Neither M-MSN-A nor M-MSN-P exhibited morphological changes after 8 h in H_2_O (data not shown) when compare to the original particles (Fig 2(a)).

In contrast to the morphological data, studies examing cellular uptake demonstrated that the nanoparticle vectors enhanced the endocytosis of CpG (Fig 5). We further verified the intracellular delivery role M-MSN-P using confocal microscopy (Fig 7). The particles were labeled by fluorescein isothiocyanate (FITC, green) and then the as-prepared delivery vehicles (M-MSN-P/CpG) were incubated with RAW264.7 cells for 3 h. Prior to live cell imaging, the nuclei were stained with 4,6-diamino-2-phenyl indole (DAPI, blue) and the endolysosomes (fusion of endosomes and lysosomes) were stained with LysoTracker Red. Therefore, the distributions of M-MSN-P and endolysosomes can be identified as green and red fluorescence, respectively. As shown in the merged image of Fig 7, the independent green dots represent nanoparticles existing within the cytoplasm, while the numerous yellow dots represent an abundant number of particles still entrapped within the endolysosomes. This confirms the successful internalization of M-MSN-Pvectors. To sum up, internalization of CpG by RAW264.7 cells depends on the extracellular release of CpG from M-MSN-P (due to the degradation of the silica shell) and the intracellular delivery by virtue of the internalized M-MSN-P.

### TNF-α secretion in RAW264.7 cells and anti-proliferative activity in Hela cells

**Fig 8:**
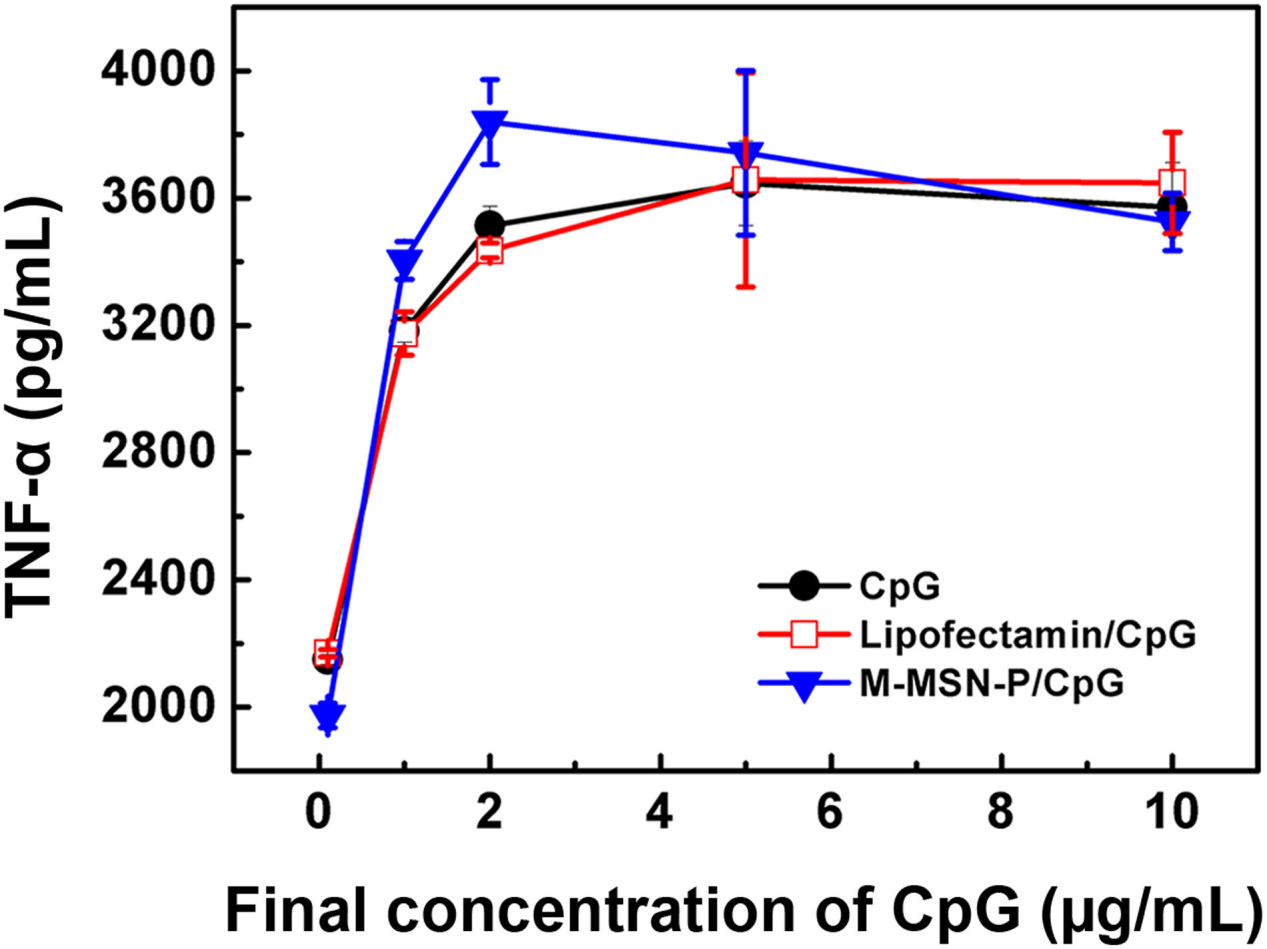
TNF-αsecretion at 8 h after the addition of CpG loaded complexes to RAW264.7 cells. Results are expressed as mean±SD of three independent measurements.

To investigate whether CpG loaded M-MSN-P are effective in activating RAW264.7 cells, TNF-α secretion was measured 8 h after the addition of CpG loaded particles. As shown in Fig 8 (Table F in S1 Text), levels of TNF-α release from cells increased with increasing concentrations of CpG for all samples following administration of low-dose drugs (final concentration of 0.1 and 1μg/ml). TNF-α secretion reached equilibrium at high concentrations of free CpG administration (>5 μg/ml) as well as administration of the other complexes examined. These results suggest that functionalized M-MSNs delivery systems are only beneficial for administration of low-dose CpG. This may be due to the rapid rise in the local concentration around the cells by virtue of the nanoparticle loading. Activation of the RAW264.7 cells by free CpG, as demonstrated by increased TNF-α secretion, is likely due to activity of the toll-like receptor-9 (TLR-9) that is essential for immuno-stimulation in the endosomes [42]; it is not necessary for CpG to escape from the endosomes or endolysosomes in order to immuno-stimulate cells. Thus, free CpG showed some immuno-stimulating effects due to the inherent phagocytic capacity of RAW264.7 cells. These results were confirmed by activation of the RAW264.7 cells by the M-MSN-P group with numerous CpG vehicles entrapped in endolysosomes (Fig 7).

**Fig 9:**
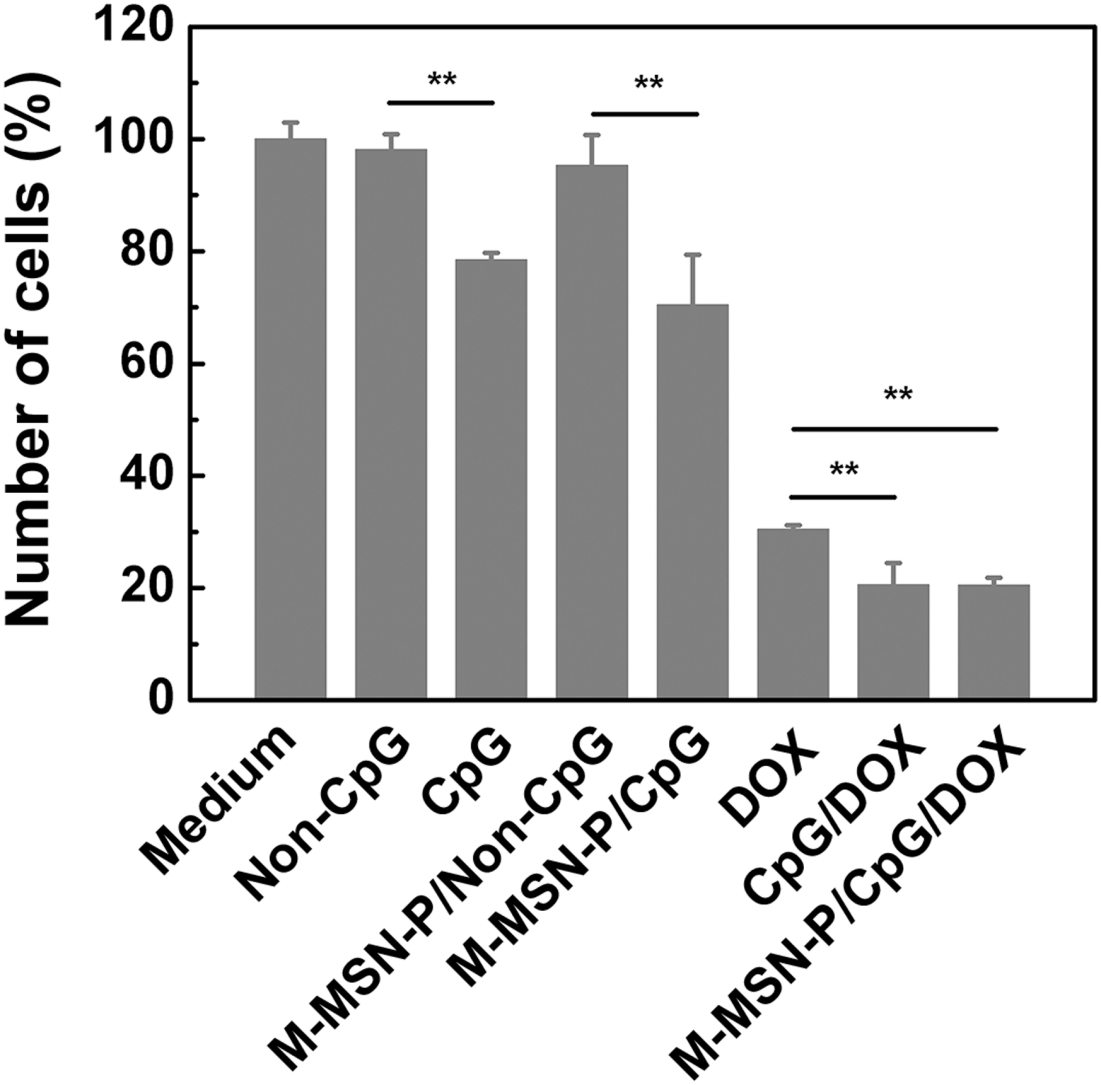
Inhibition of Hela cells proliferation by CpG loaded particles and doxorubicin hydrochloride (DOX). RAW264.7 and Hela were placed in the upper and lower chambers of Transwell plates, respectively. CpG loaded complexes and DOX were added to the upper side at a final concentration of 10 μg CpG/ml and 1 μg DOX/ml. The proliferative activity of Hela cells was measured at 48 h with the MTT assay and was converted to a percentage of the medium-treated (control) cells. Results are expressed as mean±SD of three independent measurements. CpG (and CpG loaded particle) group displayed significant differences, compared to non-CpG controls. CpG (and CpG loaded particles) also present different cell viabilities compared to the DOX treatment group (**P<0.01).

Subsequently, we measured the anti-proliferative effects of CpG loaded particles and Doxorubicin hydrochloride (denoted as DOX) on Hela cells. As shown in Fig 9 (Table G in S1 Text), both the free CpG group and CpG loaded particles group demonstrate significant growth inhibition of Hela cells (**P<0.01), compared with non-CpG controls. Similarly, all the CpG+DOX groups exhibited distinct anti-proliferative activity when compared to the DOX only treatment group (**P<0.01). These results suggest that DOX and CpG loaded systems exhibit additive effects, at a minimum, in inhibiting the proliferation of tumor cells co-cultured with RAW264.7 cells.

As mentioned above, we prepared functionalized M-MSNs (M-MSN-P) and achieved a high CpG loading capacity (180 μg/mg). However, these CpG delivery systems only showed enhanced immuno-stimulating effects when low-dose CpG (final concentration of 0.1 and 1 μg/ml) were utilized *in vitro*. Nevertheless, reports have shown that the sophisticated environment *in vivo* results in the enrichment of nanoparticles in the reticulo-endothelial system (RES) [43–45], suggesting the possibility of an increased local concentration of CpG by M-MSN-P vector loading *in vivo*. Therefore, abundant macrophages in RES would generate an intense immune effect. The nanoparticle delivery system described here may present outstanding advantages compared with free CpG *in vivo*.

### IL-12 secretion *in vivo*

**Fig 10:**
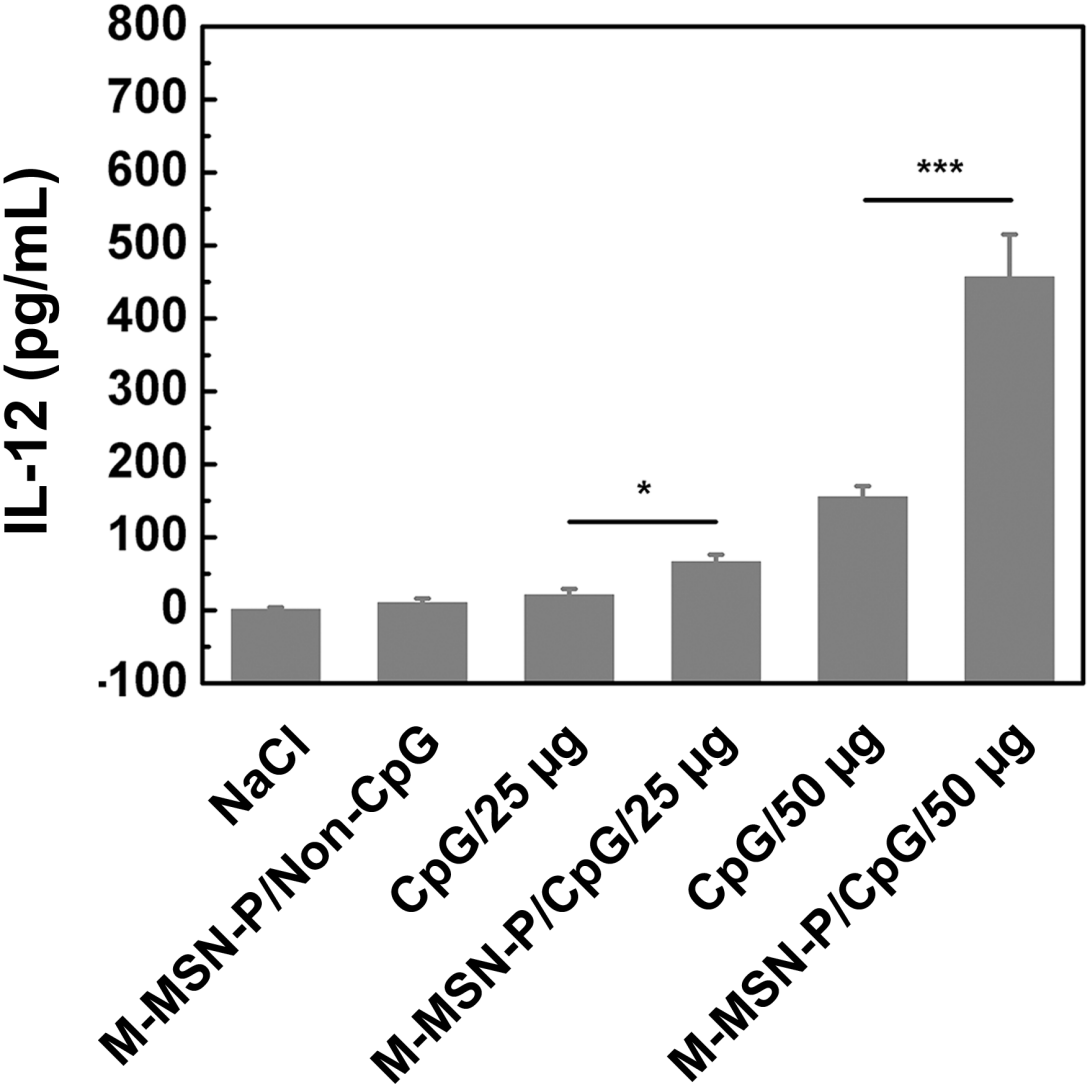
IL-12 concentration in the serum after intravenous injection of free CpG or the M-MSN-P/CpG complex. At 6 h after injection, serum samples were collected and the concentration of IL-12 was measured by ELISA. Results are expressed as mean±SD of three mice (*P<0.05, ***P<0.001).

We investigated the serum concentration of IL-12 in mice after intravenous injection of CpG loaded vehicles *in vivo*. The data are displayed in Fig 10 (Table H in S1 Text). M-MSN-P/CpG shows a significant increase in IL-12 levels compared to the free CpG group at the low dosage of 25 μg/mouse (*P<0.05). Further, the effect was more obvious when drug dosage was increased to 50 μg (***P<0.001). These results support the idea that PEGylation can increase specific particle adsorption *in vivo*, thus resulting in an increased circulatory half-life of CpG loaded within M-MSN-P, compared to free CpG [46–49]. Finally, CpG loaded particles were captured in RES and stimulated a more intense immune effect.

## Conclusion

In this study we prepared a type of CpG delivery system based on the magnetic mesoporous silica nanoparticles (M-MSNs), which were further modified by APTES and PEG (M-MSN-P). The obtained M-MSN-P posses significantly high CpG loading capacity due to appropriate size matching between its mesopores and CpG molecules. Such carriers also exhibit negligible cytotoxicity and enable to enhance the CpG internalization when incubated with the phagocytic cells. Thereby CpG therapeutics agent delivered by this kind of nanoparticles was effective in activating RAW264.7 and inhibiting tumor cells when combined with chemotherapeutics *in vitro*. Furthermore, this M-MSNs based CpG delivery systems had excellent immuno-stimulatory activity *in vivo*. To sum up, we demonstrated in this study that a kind of APTES functionalized and further PEGylated magnetic mesoporous silica nanoparticles have a true potential for use as a delivery vector for CpG ODN immunotherapies.

## Supporting Information

S1 Text Supplementary material for the manuscript (PONE-D-12-22923). (DOC)
