# Supplementary material for: Organosilane and Polyethylene Glycol Functionalized Magnetic Mesoporous Silica Nanoparticles as Carriers for CpG Immunotherapy In Vitro and In Vivo
Source: PLoS One. 2015 Oct 9;10(10):e0140265. doi: 10.1371/journal.pone.0140265 (PMC4599948; doi:10.1371/journal.pone.0140265)
Supplement: S1 Text — (DOC) [file pone.0140265.s001.doc]

**Supplementary material for the manuscript (PONE-D-15-22923)**

**Organosilane and Polyethylene Glycol Functionalized Magnetic Mesoporous Silica Nanoparticles as Carriers for CpG Immunotherapy *in vitro* and *in vivo***

Hengrui Zheng^1^, Songsong Wen^3^, Yang Zhang^4^ and Zhenliang Sun^2 *^

^1^Central for medical research, the Affiliated Hospital of Qingdao University, Qingdao, 266003, China

[[1]](#footnote-2)2Fengxian Hospital affiliated to Southern Medical University, 6600 NanFeng Road, Shanghai, 201499, China

^3^Qilu Pharmaceutical Co. Ltd, Jinan, 250101, China

^4^Tong Ren Hospital Shanghai Jiao Tong University School of Medicine，1111 XianXia Road，Shanghai 200336


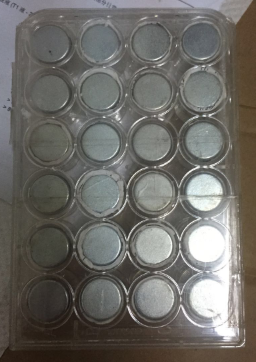


Figure A. The magnet designed specifically for 24-well cell culture plate. It can be placed under the plates for promoting the cellular uptake of magnetic nanoparticles.


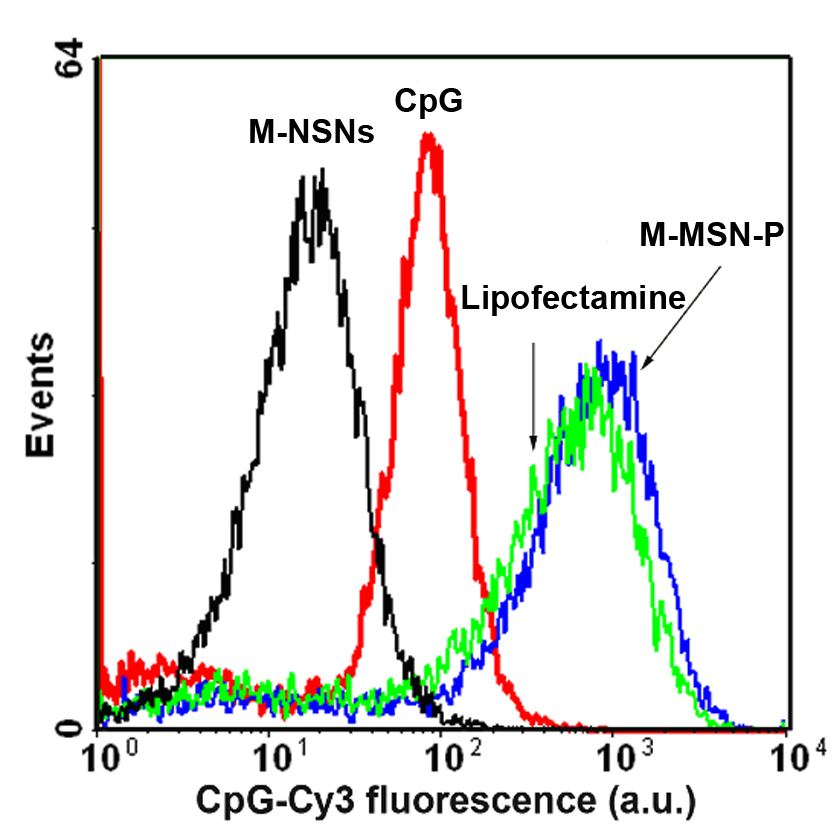


Figure B. Assessments of cell uptake by flow cytometry analysis. The cell uptake efficiency was examined by labeling the CpG with Cy-3. The free oligo CpG-Cy3 (CpG-Cy3) and Lipofectamine-CpG-Cy3 were utilized as controls. M-MSNs are unable to carry CpG-Cy3 thus no Cy3 signal is observed. M-MSN-P with CpG-Cy3 shows positive signal. The doses of free CpG or CpG in loaded carriers were equivalent to 15 µg/ml.


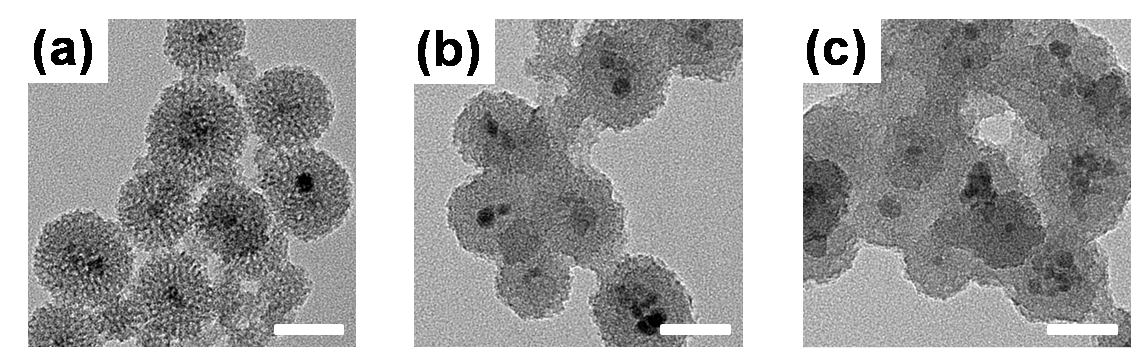


Figure C. TEM images of M-MSN-P immersed in PBS (0.1 mg/ml) for different time periods at 37 °C: (a) 1, (b) 8 and (c) 24 h, bar=50 nm.

In order to verify the stability of particles, we captured TEM micrographes of M-MSN-P after 1, 8 and 24 h immersion in PBS (0.1 mg/ml). The mixture of M-MSN-P and PBS was incubated at a constant temperature of 37 °C and shaken at a rate of 150 rpm. The data was shown in Figure B. Observed from the Figure Ca, there was no obvious structure change for M-MSN-P after an immersion time of 1 h. While the morphology of the nanoparticle composites altered 8 h later (Figure Cb), the mesoporous channels turned fuzzy and the outer surface of the particles became rough. Further, severe coalescence took place and the mesoporous structure collapsed over 24 h (Figure Cc). Combined with the particle incubation time in the cell uptake (3 h), TNF-α secretion (8 h) and TNF IL-12 production (6 h) assays, the stability of M-MSN-P should be sufficient.

**Table A. Zeta potential of M-MSN-A and M-MSN-P**

| Samples | Zeta potential (mV) |
| --- | --- |
| M-MSN-A in PB (pH 7.0) | | 34.6 | 27.6 | 42.3 | | --- | --- | --- | |
| M-MSN-P in PB (pH 7.0) | | 24.0 | 20.5 | 18.3 | | --- | --- | --- | |

**Table B. The hydrodynamic diameter analysis of M-MSN-P**

| Samples | Z-Average (d. nm) | Polydispersity Index (PDI) |
| --- | --- | --- |
| M-MSN-A in ddH_2_O | 140.1 | 0.189 |
| M-MSN-P in ddH_2_O | 146.8 | 0.169 |

Table C. CpG adsorption against M-MSN-P

| Concentration of CpG (μg/ml, before adsorption) | | | Concentration (μg/ml, after adsorption) | | |
| --- | --- | --- | --- | --- | --- |
| 41.72 | 41.17 | 41.485 | 2.53 | -0.89 | -0.89 |
| 77.75 | 78.795 | 79.105 | -0.04 | -0.58 | 0 |
| 145.67 | 146.17 | 145.87 | 5.44 | 5.29 | 4.8 |
| 257.265 | 259.47 | 259.16 | 84.6 | 84.52 | 84.3 |
| 501.035 | 500.055 | 502.65 | 324.65 | 313.86 | 324.54 |
| 613.9 | 621.685 | 622.16 | 418.26 | 413.78 | 413.47 |

Table D. OD_570 nm_ values of RAW264.7 cells incubated with particles.

| Concentration of particles in  culture medium (μg/ml) | M-MSNs | | | M-MSN-P | | |
| --- | --- | --- | --- | --- | --- | --- |
| 0 | 2.227 | 2.083 | 2.073 | 2.227 | 2.083 | 2.073 |
| 10 | 2.248 | 2.338 | 2.557 | 2.070 | 2.452 | 2.168 |
| 20 | 2.145 | 2.267 | 2.171 | 2.249 | 2.135 | 2.276 |
| 50 | 1.854 | 1.789 | 2.042 | 2.159 | 2.117 | 2.152 |
| 100 | 2.064 | 1.952 | 2.046 | 1.702 | 1.703 | 1.876 |
| 500 | 1.244 | 1.122 | 1.096 | 1.647 | 1.792 | 1.568 |
| 1000 | 0.774 | 0.750 | 0.760 | 1.544 | 1.642 | 1.442 |
| 1500 | 0.735 | 0.710 | 0.600 | 1.355 | 1.474 | 1.415 |

Table E. Concentrations of CpG in the supernatant of CpG loaded particles (μg/ml)

| Release time (h) | 0.083 | 0.5 | 1 | 2 | 4 | 8 | 12 | 24 |
| --- | --- | --- | --- | --- | --- | --- | --- | --- |
| M-MSN-A/CpG  in H_2_O | 2.68 | 7.27 | 3.61 | 5.32 | 5.21 | 8.3 | 8.85 | 10 |
|  | 1.93 | 6.69 | 3.98 | 7.81 | 6.06 | 7.99 | 9.46 | 9.17 |
|  | 2.75 | 8.72 | 4.91 | 6.22 | 6.39 | 7.7 | 8.77 | 10.28 |
| M-MSN-A/CpG  in PBS | 3.61 | 24.08 | 28.53 | 32.91 | 37.19 | 41.23 | 39.02 | 39.14 |
|  | 3.47 | 22.06 | 26.72 | 33.89 | 37.71 | 41.19 | 39.57 | 41.38 |
|  | 4.37 | 22.32 | 27.2 | 32.07 | 38.96 | 39.72 | 39.48 | 38.78 |
|  |  |  |  |  |  |  |  |  |
| Release time (h) | 0.083 | 0.5 | 1 | 3 | 6 | 12 | 24 |  |
| M-MSN-P/CpG  in H_2_O | 2.38 | 1.93 | 3.04 | 6.12 | 14.56 | 13.64 | 15.94 |  |
|  | 2.53 | 1.73 | 3.23 | 6.52 | 13.53 | 14.19 | 17.42 |  |
|  | 2.35 | 1.98 | 2.93 | 5.35 | 14.98 | 14.53 | 15.53 |  |
| M-MSN-P/CpG  in PBS | 23.43 | 28.83 | 30.93 | 27.15 | 28.39 | 30.04 | 36.13 |  |
|  | 23.13 | 29.93 | 32.32 | 27.02 | 26.31 | 32.4 | 35.23 |  |
|  | 24.28 | 30.78 | 30.42 | 26.93 | 27.97 | 31.93 | 36.83 |  |

Table F. OD_450 nm_ values of TNF-α in different samples

| Concentrations of standard samples (pg/ml) | OD _450nm_ | | Final dosage of CpG (μg/ml) | Free CpG (without vector loading)*^a^* | | Lipofectamine/CpG*^a^* | | M-MSN-P/CpG*^a^* | |
| --- | --- | --- | --- | --- | --- | --- | --- | --- | --- |
| 0 | 0.02 | 0.021 | 0.1 | 0.9 | 0.909 | 0.915 | 0.909 | 1.414 | 1.363 |
| 10.9 | 0.102 | 0.102 | 1 | 1.313 | 1.294 | 1.282 | 1.319 | 1.416 | 1.428 |
| 43.8 | 0.196 | 0.204 | 2 | 1.448 | 1.415 | 1.408 | 1.395 | 1.526 | 1.522 |
| 175 | 0.627 | 0.613 | 5 | 1.483 | 1.443 | 1.537 | 1.439 | 1.503 | 1.493 |
| 700 | 2.163 | 2.239 | 10 | 1.492 | 1.416 | 1.527 | 1.44 | 1.509 | 1.407 |

*a*: TNF-α factors were diluted 10 times when investigated

Table G. OD_570 nm_ values of Hela cells incubated with CpG loaded particles and doxorubicin hydrochloride (DOX)

| Medium | Non CpG | CpG | M-MSN-P/Non CpG | M-MSN-P/CpG | DOX | CpG/DOX | M-MSN-P/CpG/DOX |
| --- | --- | --- | --- | --- | --- | --- | --- |
| 2.903 | 2.86 | 2.286 | 2.825 | 2.054 | 0.899 | 0.656 | 0.618 |
| 3.013 | 2.817 | 2.244 | 2.798 | 1.966 | 0.894 | 0.642 | 0.6155 |
| 2.968 | 2.986 | 2.319 | 2.933 | 2.419 | 0.911 | 0.675 | 0.6215 |
| 2.816 | 2.816 | 2.325 | 2.55 | 1.801 | 0.861 | 0.433 | 0.539 |

Table H. OD_450 nm_ values of IL-12P70 in different samples

| Concentrations of standard samples (pg/ml) | OD 450 _nm_ | | Dosage of CpG (μg/mouse) | NaCl solution (0.9%) | | | Free CpG (without vector loading) | | | M-MSN-P/Non-CpG | | | M-MSN-P/CpG | | |
| --- | --- | --- | --- | --- | --- | --- | --- | --- | --- | --- | --- | --- | --- | --- | --- |
| 0 | 0.125 | 0.110 | 25 | 0.142 | 0.135 | 0.144 | 0.167 | 0.156 | 0.172 | 0.153 | 0.155 | 0.145 | 0.182 | 0.173 | 0.202 |
| 78.125 | 0.254 | 0.234 |  |  |  |  |  |  |  |  |  |  |  |  |  |
| 156.25 | 0.312 | 0.339 | 50 |  |  |  | 0.326 | 0.342 | 0.306 |  |  |  | 0.462 | 0.559 | 0.486 |
| 312.5 | 0.554 | 0.533 |  |  |  |  |  |  |  |  |  |  |  |  |  |
| 625 | 0.856 | 0 |  |  |  |  |  |  |  |  |  |  |  |  |  |

1. ^2*^ Corresponding author. Tal/fax.: +86 21 57423899

   Hengrui Zhen and Songsong Wen contributed equally to this work and worked as co-first authors.

   E-mail address: [hope1126@hotmail.com](mailto:hope1126@hotmail.com) (Z. Sun). [↑](#footnote-ref-2)
